# Determinants of low fifth minute Apgar score among newborns delivered by cesarean section at Wolaita Sodo University Comprehensive Specialized Hospital, Southern Ethiopia: an unmatched case control study

**DOI:** 10.1186/s12884-022-04999-z

**Published:** 2022-08-26

**Authors:** Bahiru Darma Ajibo, Eskinder Wolka, Andualem Aseffa, Mitiku Ayele Nugusu, Abdi Oumer Adem, Mebratu Mamo, Ashagrie sintayehu Temesgen, Getachew Debalke, Negeso Gobena, Mohammad Suleiman Obsa

**Affiliations:** 1 School of Anesthesia, Wolaita Sodo University, Wolaita Sodo, Ethiopia; 2 Departments of Anesthesia, Arsi University, Asella, Ethiopia; 3 Department of Anesthesia, Hawassa University, Hawassa, Ethiopia; 4 Schools of Public Health, Wolaita Sodo University, Wolaita Sodo, Ethiopia

**Keywords:** Associated factors, Apgar score, Southern Ethiopia, Unmatched case control

## Abstract

**Background:**

Apgar score is used to evaluate the neonates’ overall status and response to resuscitation, as well as its prognosis beyond the neonatal period. Low fifth minute Apgar scores is more frequent and is associated with markedly increased risks of neonatal mortality and morbidity. In Ethiopia, the prevalence of birth asphyxia is high (22.52%). Birth asphyxia contributes to significant neonatal morbidities and mortalities due to severe hypoxic-ischemic multi-organ damage, mainly brain damage. Therefore, this study was aimed to identify determinants of low fifth minute Apgar score among newborns delivered by cesarean section.

**Methods:**

An unmatched case control study design was conducted. The Apgar score is based on measures of heart rate, respiratory effort, skin color, muscle tone, and reflex irritability. The data collection tool or checklist was adapted from previous study done at Addis Ababa, Ethiopia. In this study, cases were all newborns with Apgar score < 7 whereas controls were all newborns with Apgar score >  = 7. The study participants were selected by simple random sampling technique. Data was into Epidata version 4.6 and exported to SPSS software version 24. Multivariable logistic regression was used to identify the independent effect of different factors at *P* < 0.05.

**Result:**

Factors associated with low Apgar score were fetal birth weight < 2.5 kg [adjusted odds ratio (AOR) = 8.17, 95% confidence interval (CI): 1.03 ‒ 64.59] *P* = 0.046, skin incision to delivery time (AOR = 5.27; 95% CI: 2.20 ‒ 12.60) *P* = 0.001, pregnancy induced hypertension (AOR = 4.58, 95% CI: 1.75 ‒ 11.92) *P* = 0.002, antepartum hemorrhage (AOR = 3.96; 95% CI: 1.75 ‒ 8.94) 0.001, general anesthesia (AOR = 3.37, 95% CI: 1.72 ‒ 6.62) *P* = 0.001, meconium stained amniotic fluid (AOR = 3.07, 95% CI: 1.32 ‒ 7.12) *P* = 0.009 and emergency cesarean section (AOR = 2.17, 95% CI: 1.13 ‒ 4.15) *P* = 0.019.

**Conclusions:**

Fetal birth weight < 2.5 kg, skin incision to delivery time, pregnancy induced hypertension, antepartum hemorrhage, type of anesthesia, meconium stained amniotic fluid and type of cesarean section were factors independently associated with Apgar score. Therefore, it is important to work on identified risk factors to reduce the impacts low fifth minute Apgar score in the in early adulthood..

**Supplementary Information:**

The online version contains supplementary material available at 10.1186/s12884-022-04999-z.

## Introduction

The Apgar score was proposed in 1952 as a means of rapidly evaluating the clinical status of newborn infants and currently remains an accepted method for newborn infant assessment immediately after delivery [1]. Apgar score was estimated using five variables: strength and regularity of heart rate, lung maturity or breathing effort, muscle tone and movement, skin color or oxygenation and reflex response to irritable stimuli. The Apgar score ranges from 0 to 10. A total score of 7‒10 is considered as “normal” while a total score of below 7 is considered as “low Apgar score”. a lower Apgar score indicates depressed vitality [2].

Apgar score is usually done to the baby twice: once at first minute after birth, and again at fifth minute after birth. The first minute Apgar score may signal the need for immediate resuscitation, and the 5 min score may signal the probability of successfully resuscitation following the record the first minute Apgar score. This means that if there is successfully resuscitation at the first minute, the Apgar score at fifth minute will be satisfactory. On the other hand, a low Apgar score at 1 min may not necessarily mean a low Apgar score at 5 min [3, 4]. The 5 min Apgar score may also provide an indication of a neonate’s sustained capacity to survive and thrive [5]. It is for this reason that the second Apgar score assessment, made at 5 min, is a better predictor of later outcomes than the Apgar score at first minute [6].

In Africa, birth asphyxia is responsible for 24.0% of the newborn deaths; and in Sub-Saharan Africa, birth asphyxia is accounted for 280,000 neonatal deaths [7]. An umbrella review of systematic review and meta-analysis conducted on the state of birth asphyxia in Ethiopia shows the prevalence of birth asphyxia is as high as 22.52%. Birth asphyxia contributes to significant neonatal morbidities and mortalities due to severe hypoxic-ischemic multi-organ damage, mainly brain damage [8].

Many studies have demonstrated that 5 min Apgar score increases the risk for later motor control and perception difficulties, cognitive developmental delays, learning disabilities, cerebral palsy, autism, attention deficit or hyperactivity disorder, and epilepsy [9]. Another study also showed that neonates with low Apgar score at 5 min has an increased risk is of neonatal respiratory distress, need for mechanical ventilation and admission to neonatal intensive care unit [10], and also, a higher risk of childhood cancers [11]. In our setting, significant attention has been given to Apgar score at birth rather than at fifth minute despite of its paramount long term sequel. Therefore, identifying determinants of low fifth minute Apgar score is very important to reduce the long term complication during the phase of early Childhood.

## Methods and materials

This unmatched case control study was conducted at Wolaita Sodo University Comprehensive Specialized Hospital, Southern Ethiopia. The hospital is found in Sodo town which is located about 329 km from Addis Ababa, capital city of Ethiopia. The Sodo town is also located about151 kilometers from Hawassa, the capital city of southern nations nationalities peoples regional state. It has four major and minor departments of which Obstetrics and Gynecology is one of the major departments. Annually, more than 1000 Caesarian section could be done at the hospital.

In this study, all charts of pregnant mothers who gave births by caesarian section at Wolaita Sodo University Comprehensive Specialized Hospital were source population whereas all complete two years (September 01, 2017 to September 01, 2019 G.C) charts of pregnant mothers gave birth by caesarian section at Wolaita Sodo University Comprehensive Specialized Hospital and which fulfilled the inclusion criteria were our study population. The charts which had no the record of 5 min Apgar score, charts with incomplete documentation (more than three missing value), deliveries of unknown gestational age and newborns with documented gross congenital anomalies were excluded. In this study, cases were all charts of newborns with Apgar score < 7 whereas controls all charts of newborns with Apgar score >  = 7. Preeclampsia was used to calculate the final sample size as it gave the maximum sample size 329 [110 cases and 219 controls] with the following assumptions [12], 95% confidence interval, 80% power, 2.37 odds ratio, 2:1 controls to case ratio. Before selecting cases and controls, all two years charts of pregnant mothers were identified. Then, charts which contained Apgar score greater than or equal to 7 and less than 7 were placed into two separate groups. After that, simple random sampling technique was used to separately select both cases and controls from its respective group. The Apgar score was recorded on the chart based on the appearance (color), pulse rate, grimace (reflexes), muscle tone (activity) and respiratory effort of neonate. Each component carries a score from 0 to 2 (Table 1). It was done as per the protocol of neonatal advanced life support advocated by the American pediatric association (APA) [13].

**Table 1:** Apgar score scale of measurement

Apgar Score	0	1	2
Heart rate	Absent	> 100	> 100
Respiratory effort	Absent	Irregular	Good
Reflex irritability	No response	Reflex irritability	Cough/ sneeze
Appearance (color)	Blue or pale	Body pink with blue extremities	Completely pink
Muscle tone	Flaccid	Good tone	Spontaneous flexion

The data collection tool or checklist was adapted from previous study done at Addis Ababa, Ethiopia [14] and then modified and used in the current study. Four undergraduate anesthesia students and two masters of Science degree anesthesia students were recruited as data collectors and supervisors respectively. Training was given for the data collectors and supervisors for one day. The aim of the training was to make understanding on the objective of the study, data collection tool, data quality assurance and data collection procedures. Pre-test was carried out on 5% of sample size at Arba Minch general hospital. After pretest was undertaken, three exploratory variables (educational level, monthly income and occupation) were omitted from the checklist. The collected data was checked for completeness and consistencies on a daily basis. Then, it was entered into Epi data version 4.6 and exported to SPSS software version 24 for analysis. A binary logistic regression was conducted to identify the association between the dependent and independent variables. All variables with *P*—value less than 0.25 in the binary logistic regression were included in the multivariable logistic regression. Chi-square test was used to compare categorical variables between groups. The multicollinearity among independent variables was checked by variance inflation factor (VIF) and tolerance test and it was found that there was significant correlation between independent variables. AOR with 95% CI and *P* < 0.05 were used to measure strength of association. Model adequacy was checked by the non-significant hosmer and lameshow goodness of fit test (*P* = 0.673).

## Results

### Personal and socio‒demographic characteristics of study participants

A total of 329 participants (110 cases and 219 controls) were included in this study. The mean ages of the mothers of cases and controls were 26.8 (SD = 4.3) and 26.7 (SD = 4.7) years, respectively. 50(45.5%) mothers of cases and 88(40.2%) controls were with in age group 25‒29 years. Age group <  = 19 years and >  = 30 years comprises 2(1.8%) and 33(30.0%) for cases and 6(2.7%) and 67 (30.6%) for controls. Majority of mothers of the cases 100(90.9%) and controls 181(82.6%) groups were in the ethnic group of Wolaita. More than half, 60(54.5%) of cases and 140(63.9%) controls were urban residents. Neither cases nor controls have smoking or alcohol drinking history (Table 2).

**Table2:** Personal and socio-demographic factors of mothers who gave birth by CS at Wolaita Sodo University Comprehensive Specialized Hospital, Southern Ethiopia Southern Ethiopia from January 1 to December 30, 2019

Variables	Category	Cases (*n* = 110)	Controls (*n* = 219)	Total (%) (*n* = 329)	*P* -Value
		Frequency (%)	Frequency (%)		
Age	< = 19	2(1.8)	6(2.7)	8(2.4)	.763
	20–24	25(22.7)	58(26.5)	83(25.2)	
	25–29	50(45.5)	88(40.2)	138(41.9)	
	> = 30	33(30.0)	67(30.6)	100(30.4)	
Ethnicity	Wolaita	100(90.9)	181(82.6)	281(85.4)	.132
	Hadiya	3(2.7)	13(5.9)	16(4.9)	
	Gamo	7(6.4)	25(11.4)	32(9.7)	
Residence	Urban	60(54.5)	140(63.9)	200(60.8)	.100
	Rural	50(45.5)	79(36.1)	129(39.2)	

### Obstetrics, surgical or anesthesia related factors

Among the total participants more than half, 62 (56.4%) mothers of the cases and 117 (53.4%) of the controls were multigravida while 48 (43.6%) mothers of the cases group and 102 (46.6%) mothers of the controls group were primigravida. Twenty six (23.6%) mothers in cases group and 37 (16.9%) mothers in controls group had premature rupture of membrane (PROM). Regarding the cord prolapse, 13(11.8%) mothers in cases group and 14(6.4%) mothers in controls group had cord prolapse; 22 (20.0%) mothers of the cases and 19 (8.7%) of the controls had ante partum hemorrhage during pregnancy. This study has shown that majority of mothers 103 (93.6%) of cases and 196 (89.5%) of controls had ever got ANC service whereas 7 (6.4%) of cases and 23 (10.5%) of controls had never got ANC service during their pregnancy of the current neonate. Twenty one (19.1%) mothers in cases group and 14 (6.4%) mothers in controls group had meconium stained amniotic fluid (MSAF); 30 (27.3%) of the cases and 42 (19.2%) of the controls were born from mothers with history of prolonged second stage of labor. The proportion of women with pregnancy‒induced hypertension was higher in cases 20 (18.2%) than controls 13 (5.9%) (Table 3).

**Table 3:** Obstetrics, surgical or anesthesia related factors of low fifth minute Apgar score among newborns delivered by Caesarian section at Wolaita Sodo University Comprehensive Specialized Hospital, Southern Ethiopia Southern Ethiopia from January 1 to December 30, 2019

Variables	Category	Cases (*n* = 110)	Controls (*n* = 219)	Total (%) (*n* = 329)	*p*-value
PROM	Yes	26(23.6)	37(16.9)	63(19.1)	.143
	No	84(76.4)	182(83.1)	266(80.9)	
Cord prolapse	Yes	13(11.8)	14(6.4)	27(8.2)	.091
	No	97(88.2)	205(93.6)	302(91.8)	
Antepartum hemorrhage	Yes	22(20.0)	19(8.7)	41(12.5)	.003*
	No	88(80.0)	200(91.3)	288(87.7)	
ANC follow up	Yes	103(93.6)	196(89.5)	299(90.9)	.219
	No	7(6.4)	23(10.5)	30(9.1)	
MSAF	Yes	21(19.1)	14(6.4)	35(10.6)	.001*
	No	89(80.9)	205(93.6)	294(89.4)	
prolonged second stage of labor	Yes	30(27.3)	42(19.2)	72(21.9)	.094
	No	80(72.7)	177(80.8)	257(78.1)	
Pregnancy induced hypertension	Yes	20(18.2)	13(5.9)	33(10.0)	.001*
	No	90(81.8)	206(94.1)	296(90.0)	
	No	108(98.2)	215(98.2)	323(98.2)	
	No	107(97.3)	217(99.1)	324(98.5)	
Type of anesthesia	Spinal	78(70.9)	189(86.3)	267(81.2)	.001*
	General	32(29.1)	30(13.7)	62(18.8)	
Type of caesarian section	Elective	71(64.5)	123(56.2)	194(59.0)	.001*
	Emergency	39(35.5)	96(43.8)	135(41.0)	
Incision to delivery time	< = 3	84(76.4)	209(95.4)	293(89.1)	.001*
	> 3	26(23.6)	10(4.6)	36(10.9)	
Induction to delivery time	< = 6	71(64.5)	132(60.3)	203(61.7)	.452
	> 6	39(35.5)	87(39.7)	126(38.3)	
Sex of newborn	Male	60(54.5)	123(56.2	183(55.6)	.780
	Female	5045.5)	96(43.8)	146(44.4)	
Type of gestation	Single	108(98.2)	213(97.3)	321(97.6)	.464
	Multiple	2(1.8)	6(2.7)	8(2.4)	
Gestational age	Preterm	2(1.8)	14(6.4)	16(4.9)	.232
	Term	108(98.2)	200(91.3)	308(93.6)	
	Post term	0(0.00)	5(2.3)	5(1.5)	
Birth weight	< 2.5	1(.9)	19(8.7)	20(6.1)	.021*
	2.5–4.0	99(90.0)	182(83.1)	281(85.4)	
	> 4.0	10(9.1)	18(8.2)	28(8.5)	
Fetal heartbeat	< 120	4(3.6)	7(3.2)	11(3.3)	.084
	120–160	103(93.6)	189(86.3)	292(88.8)	
	> 160	3(2.7)	23(10.5)	26(7.9)	

^*^ = statistical significant at *p*-value < 0.05

### Determinants of Apgar score

The bivariate analysis showed that pregnancy induced hypertension, antepartum hemorrhage, ANC follow up, Cord prolapse, prolonged second stage of labor, PROM, MSAF, fetal birth weight, type of anesthesia, type of caesarian section and incision to delivery time more than 3 min were significantly associated with low fifth minute Apgar score.

The multivariable analysis result showed that the likelihood of developing low Apgar score among neonates born from mothers with pregnancy induced hypertension was 4.58 times (AOR = 4.58, 95% CI, 1.75‒11.92) more compared with their counter‒parts. Neonates born with MSAF fluid were 3.07 times (AOR = 3.07, 95% CI: 1.32 ‒ 7.12) as likely to have low Apgar score as those born without being meconium stained. Neonates born with low birth weight < 2.5 kg were 8.17 times (AOR = 8.17, 95% CI: 1.03 ‒ 64.59) as likely to have low Apgar score compared to those born with normal weight. Regarding the APH, newborns delivered from mothers with Antepartum hemorrhage were approximately four times (AOR = 3.96, 95% CI: 1.75 ‒ 8.94) more likely to develop low Apgar score compared to their counter parts. Moreover, the likelihood of encountering low Apgar score was 3.37 times (AOR = 3.37; 95% CI: 1.72 ‒ 6.62) higher among neonates born under general anesthesia compared to spinal anesthesia [15]. In this study, the higher odds of developing low Apgar score was also observed among neonates born by emergency CS compared to elective CS (AOR = 2.17;95% CI: 1.13 ‒ 4.15). Similarly, newborn with skin incision to delivery time above three minutes were prone to low Apgar score with an odds ratio of 5.27(AOR = 5.27; 95% CI: 2.20‒12.60). (Table 4) shows bivariate and multivariate analysis of determinant factors of low Apgar score among newborns delivered by CS at WSUCSH from January 1to December 30, 2019.

**Table 4:** Bivariable and multivariable results of factors associated with low fifth minute Apgar score among newborns delivered by Caesarian section at Wolaita Sodo University Comprehensive Specialized Hospital, Southern Ethiopia from January 1 to December 30, 2019

Variables	Category	Cases N(%)		Controls N(%)		COR(95%CI)	*P*‒value	AOR(95%CI)	*P*‒value
Incision time	> 3	26	23.6	10	4.6	1	1	1	
	< = 3	84	76.4	209	95.4	6.46(2.98‒14.00)	.000	5.27(2.20‒12.60)	.001*
Pregnancy induced hypertension	Yes	20	18.2	13	5.9	3.52(1.67‒7.38)	.001	4.58(1.75‒11.92)	.002*
	No	90	81.8	206	94.1	1	1	1	
Cord prolapse	Yes	13	11.8	14	6.4	1.96(.88‒4.33)	.095	1.93(0.77‒4.84)	.095
	No	97	88.2	205	93.6	1	1	1	
Type of caesarian section	Elective	71	64.5	123	56.2	1	1	1	
	Emergency	39	35.5	96	43.8	1.42(.88‒2.28)	.146	2.17(1.13‒4.15)	.019*
PSSL	Yes	30	27.3	42	19.2	1.58(.92‒2.70)	.095	1.54(0.82‒2.88)	.170
	No	80	72.7	177	80.8	1	1	1	
PROM	Yes	26	23.6	37	16.9	1.52(.86‒2.67)	144	1.32(0.68‒2.57)	.398
	No	84	76.4	182	83.1	1	1	1	
Antepartum hemorrhage	Yes	22	20.0	19	8.7	2.63(1.35‒5.10)	.004	3.96(1.75‒8.94)	.001*
	No	88	80.0	200	91.3	1	1	1	
Type of anesthesia	General	32	29.1	30	13.7	2.58(1.47‒4.54)	.001	3.37(1.72‒6.62)	.001*
	Spinal	78	70.1	189	86.3	1	1	1	
MSAF	Yes	21	19.1	14	6.4	3.45(1.68‒7.10)	.001	3.07(1.32‒7.12)	.009*
	No	89	80.9	205	93.6	1		1	
Birth weight	< 2.5	1	.9	19	8.7	10.33(1.36‒78.36)	.024	8.17(1.03‒64.59)	.046*
	2.5‒4	99	90.0	182	83.1	1	1	1	
	> 4	10	9.1	18	8.2	.979(.43‒2.20)	0.959	1.50(.58‒3.85)	.400
ANC follow up	Yes	103	93.6	196	89.5	1.72(.71‒4.15)	.223	2.72(.91‒8.06)	.07
	No	7	6.4	23	10.5	1	1	1	

^*^ = statistical significant at *p*-value < 0.05

## Discussion

The current study was conducted to identify determinants of low fifth minute Apgar score among newborns delivered by caesarian section. In this study: pregnancy induced hypertension, type of caesarian section, antepartum hemorrhage, type of anesthesia, incision to delivery time, meconium stained amniotic fluid and low birth weight (< 2.5 kg) were found to be significantly associated with low Apgar score.

In this study, we did not identify the prevalence of low fifth minute Apgar score or birth asphyxia because of the nature our study design (case controls study design). However, a cross-sectional study done on birth asphyxia and associated factors among newborns delivered in Jimma Zone Public Hospitals, Southwest Ethiopia shows prevalence of birth asphyxia was 32.9% in the first and 12.5% in the fifth minute [16]. Similarly, other studies done in public hospital in northeast Amhara [17] and at Debre Tabor General Hospital, North Central Ethiopia [18] revealed that 22.6% and 28.35% of all newborn had birth asphyxia respectively. This variation might be due to differences in sample size and study setting.

Type of anesthesia was significantly associated with the risk of developing low Apgar score, accordingly new born whose mothers underwent general anesthesia [AOR = 3.37;95% CI(1.72‒6.62)] showed significant association with low Apgar score, as compared with new born whose mothers underwent spinal anesthesia. This finding is consistent with the study conducted at Arbaminch general hospital, southern Ethiopia [19] and Abbasi Shaheed hospital, Pakistan [20]. This could be explained by anesthetic drugs given during general anesthesia cross the placenta and cause fetal depression.

Those newborns delivered from mothers who underwent emergency caesarian section were about 2.17 times (AOR = 2.17; 95% CI: 1.13 ‒ 4.15) more likely to have low Apgar score than those delivered from mothers whom underwent elective caesarian section. This finding was supported by study conducted at Rabat, Morocco [21]. This might be due to the fact that either most of the mothers come with complications or the decision to for caesarian section might be late after they develop complications.

A data from multivariate analysis also showed that babies born when skin incision to delivery time was above three minutes was 5.27 times (AOR = 5.27; 95% CI: 2.20‒12.60) more likely to have low Apgar score compared to those with skin incision to delivery time <  = 3. This is in line with study conducted at Gandhi memorial hospital, Ethiopia [14]. The low Apgar score when skin incision to delivery time is prolonged might be due to decrease in blood pressure because of supine hypotension syndrome or effect of anesthetic drugs. This causes decrease in uteroplacental circulation and causes low Apgar score.

Meconium stained amniotic fluid had also a significant association with the occurrence of low Apgar score. Specifically, newborns delivered with meconium stained amniotic fluid were 3.07 times (AOR = 3.07, 95% CI: 1.32 ‒ 7.12) more likely to develop low Apgar score compared with that of their counter parts. This finding is compatible with studies from Gonder, Northwest Ethiopia [22] and India [23]. This might be due to the reason, newborns delivered from mothers with meconium stained amniotic fluid are more predisposed to aspirate it and fill smaller airways and alveoli in the lung. This can lead to airway obstruction and limited lung movement. Then due to poor gas exchange birth asphyxia evidenced by low Apgar score could occur.

This study revealed that neonates born to mothers with PIH were 4.58 times more likely to have LAS than their counterparts [AOR = 4.58:95% CI (1.75‒11.92)]. This is in line with studies done in Lemlem Karl general hospital, Northern Ethiopia [15], Sweden and India [24, 25]. This might be due to, PIH causes placental ischemia. Placenta may bleed or it may begin to separate from the wall of the uterus prematurely. This cause limited transfer of oxygen or nutrients to the fetus. If any of these complications occurs, fetal distress may develop ultimately leads to low Apgar score.

Odds of low Apgar score increased about 3.96 times more comparing newborns from mothers with APH to those who do not have the event (AOR = 3.96 95%CI:1.75‒8.94). This is in line with studies conducted at Addis Ababa, Ethiopia [26] and Tigray Ethiopia [27]. The possible explanation could be because of the consequence of antepartum bleeding. If APH occurs there will be decreased blood flow from mother to fetus through placenta, so that the hypoxemia can occur in the fetus. This condition can lead to birth asphyxia; this in turn leads to low Apgar score.

Moreover, this study also revealed newborns born with low birth weight were eight times more likely to have low Apgar score AOR = 8.17(95% CI:1.03‒64.59). This study is agreeable with studies done at Asella teaching referral hospital, south eastern Ethiopia [28] and Pakistan [29]. This could be explained by the fact that small babies might suffer difficult breathing and might develop difficulty in cardiopulmonary transition and perinatal asphyxia which predisposes the newborns to different complications including low fifth minute Apgar score.

### Limitations of the study

The main limitations of this study were: there is information bias due to poor recording and post-delivery factors which may affect Apgar score were not considered.

## Conclusion

The findings of this study showed that skin incision to delivery time, pregnancy induced hypertension, antepartum hemorrhage, type of anesthesia, type of caesarian section, birth weight and presence of MSAF were factors significantly associated with low Apgar score. Therefore, is very important to work on identified risk factors so as to reduce the future complication of low fifth minute Apgar score.

## Supplementary Information

**Additional file 1.**

## Data Availability

The datasets used and/or analyzed during the current study are available from the corresponding author on reasonable request.

## Abbreviations

ANC: Ante Natal Care
COR: Crude Odds Ratio
