# Supplementary material for: Determinants of low fifth minute Apgar score among newborns delivered by cesarean section at Wolaita Sodo University Comprehensive Specialized Hospital, Southern Ethiopia: an unmatched case control study
Source: BMC Pregnancy Childbirth. 2022 Aug 26;22:665. doi: 10.1186/s12884-022-04999-z (PMC9413889; doi:10.1186/s12884-022-04999-z)
Supplement: Supplementary file 1 — Additional file 1. [file 12884_2022_4999_MOESM1_ESM.pdf]

ሳ/ከ/ት/ሚኒስቴር
ወላይታ ሶዶ ዩኒቨርሲቲ
University
የጤና ሳይንስና የህክምና ኮሌጅ
ወላይታ ሶዶ

MOSHE
Wolaita Sodo
College of Health Sciences & Medicine
Wolaita Sodo

Chief Research and Community service Director Office
ቺፍ ምርምርና ማህበረሰብ አገልግሎት ዳይሬክቶሬት ጽ/ቤት

ቁጥር/Ref.No CRCS-D 65/01/13

ቀን/Date 29 - 11 - 13

Title of the project: Determinants Of Apgdar Score Among Newborns Delivered By Cesearean Section At Wolaita Soddo University Teaching And Referral Hospital, Soddo, Ethipopia, 2021: Unmatched Case Control Study .

Principal investigator: Mr. Bahiru Darma

Project No. CHSM/ERC/8/13

Recommendation of College of Health Science WSU Ethical review committee

The request for an initial review on the above mentioned project was duly considered and approved by the ERC of College of Health Sciences during its meeting held on Nov 18, 2021. The investigator should submit final report to the college up on completion. The investigator should also notify ERC ahead of any amendment or modification of the protocol or premature suspension or termination of the study

Kind regards,

ባንቺዓለም ነጋ አንጎሬ
Banchialem Nega Angore
ቺፍ ምርምርና ማህበረሰብ [illegible] ዳይሬክተር
Chief Research &
Community Service Director

C/C

- Chief Research Community Service Director
- Mr. .Bahiru Darma

Otona,
